# Arthroscopically Assisted Evacuation of Brodie’s Abscess of Distal Femur

**DOI:** 10.7759/cureus.959

**Published:** 2017-01-06

**Authors:** Rajeev R Manandhar, Shisir Lakhey, Sagar Panthi, Kiran P Rijal

**Affiliations:** 1 Orthopaedics, Kathmandu Medical College; 2 Orthopaedics and Trauma Surgeon, Nepalgunj Medical College and Teaching Hospital

**Keywords:** brodie’s abscess, distal femur, arthroscopic assisted drainage

## Abstract

Brodie’s abscess is a type of subacute osteomyelitis. Opinions differ as to whether treatment should be surgical or medical for these classic lesions. Failure of symptoms to resolve after six weeks of antibiotics or worsening of the condition during treatment should be followed by surgical treatment. Clinical signs of subperiosteal pus or synovitis indicate that the subacute infection has transformed into an acute component, and it must be drained surgically. Surgical treatment is comprised of evacuation and curettage for small lesions and evacuation, packing with cancellous bone chips, for large cavities. When clinical signs of synovitis are present, with a possibility of pus within a joint, arthrotomy is performed. Arthroscopically assisted evacuation of Brodie’s abscess from the distal femur has never been reported in the literature.

We report a case of a 23-year-old female who presented with pain and swelling over the left knee for four months. There was diffuse swelling in the knee; tenderness was present over medial femoral condyle and range of motion (ROM) of the knee was five to 45 degrees at the time of presentation. X-ray and magnetic resonance imaging (MRI) revealed Brodie’s abscess on the lateral aspect of the medial femoral condyle. The patient was treated with the evacuation of pus and curettage of the cavity using an arthroscope. After two weeks, the patient had mild pain with knee ROM from zero to 45 degrees, and at the one-month follow-up, the knee ROM improved to zero to 90 degrees. At the two-year follow-up, the patient had no pain, with knee ROM from zero to 120 degrees.

## Introduction

Subacute osteomyelitis is a distinct form of osteomyelitis, and Brodie’s abscess is one of the subtypes. Diagnosis of Brodie’s abscess is often difficult because the characteristic signs and symptoms of the acute form of the disease are absent [1-3]. In non-contemporary literature, Brodie’s abscess was referred to as a chronic form of osteomyelitis; however, in almost all contemporary literature references, Brodie’s abscess is known as the most common type of subacute osteomyelitis.

Sir Benjamin Brodie, a surgeon at St. George's Hospital, London, United Kingdom, first described subacute osteomyelitis in 1832 [4]. Brodie found a walnut-sized cavity filled with dark-colored pus. The bone immediately surrounding the cavity was whiter and harder than the surrounding bone. The inner surface of the cavity appeared to be highly vascular. Since then, low-grade pyogenic abscesses of the bone have frequently been referred to as Brodie’s abscess. The lower limb is affected more often than the upper limb, and the tibia more than the femur [5].

The treatment of Brodie’s abscess is divided into medical management and surgical management. Failure of symptoms to resolve after six weeks of antibiotics or worsening of the condition during treatment should be followed by surgical treatment. Surgical treatment is comprised of evacuation and curettage for small lesions and evacuation, packing with cancellous bone chips, for large cavities. We have managed a 23-year-old female patient suffering from Brodie’s abscess of the left distal femur with arthroscopically assisted evacuation.

Informed consent was obtained from the patient, and IRB approval was provided by Kathmandu Medical College Review Board (approval #1977).

## Case presentation

**Figure 1:**
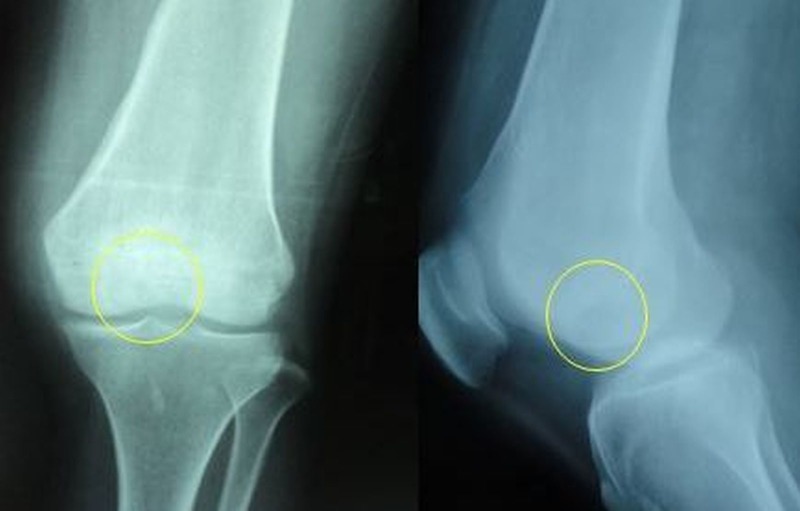
Anteroposterior and lateral radiograph showing the lytic lesion involving the lateral aspect of the medial femoral condyle

A 23-year-old female patient came to our orthopedic clinic with chief complaints of pain and swelling in her left knee for four months. The pain on the left knee was gradual in onset, progressive in nature, and dull aching in character. Pain usually increased at night, was aggravated by walking, and was relieved by rest. On examination, there was antalgic gait with diffuse swelling over left knee, and tenderness was present over medial condyle of left femur. Knee ROM was five to 45 degrees. Anteroposterior and lateral view radiographs of the knee were obtained (Figure 1).

**Figure 2:**
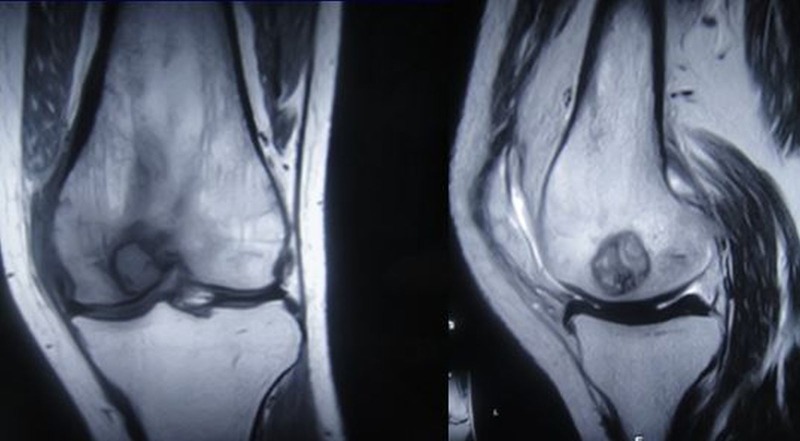
MRI showing increased intensity of the lesion

**Figure 3:**
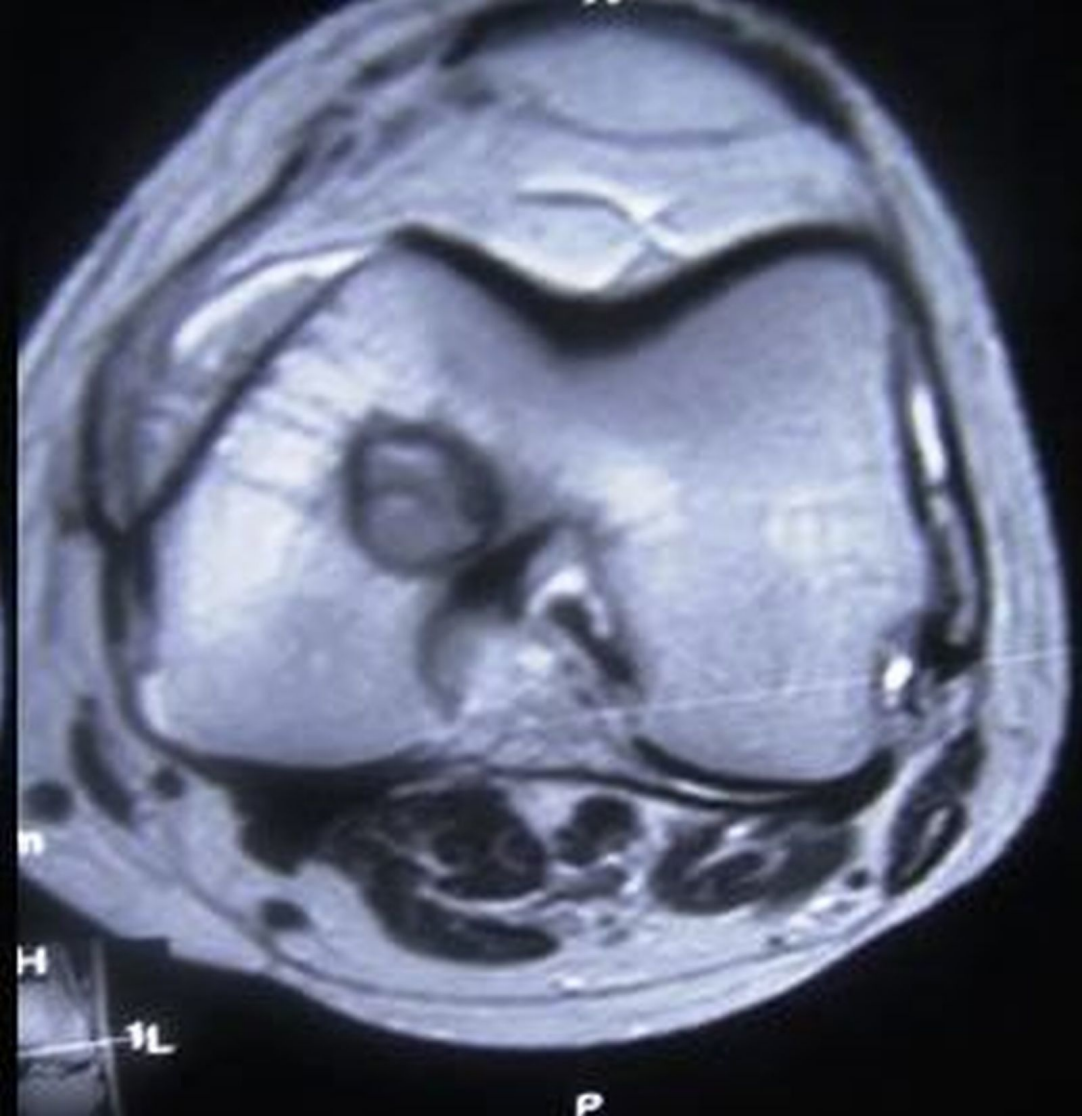
MRI axial section showing increased signal intensity of the lesion

Radiographs revealed a small radiolucent lesion on the lateral aspect of the medial femoral condyle of the distal femur. MRI also showed a well-defined lesion of increased signal intensity on the lateral aspect of the medial femoral condyle (Figures 2-3).

**Figure 4:**
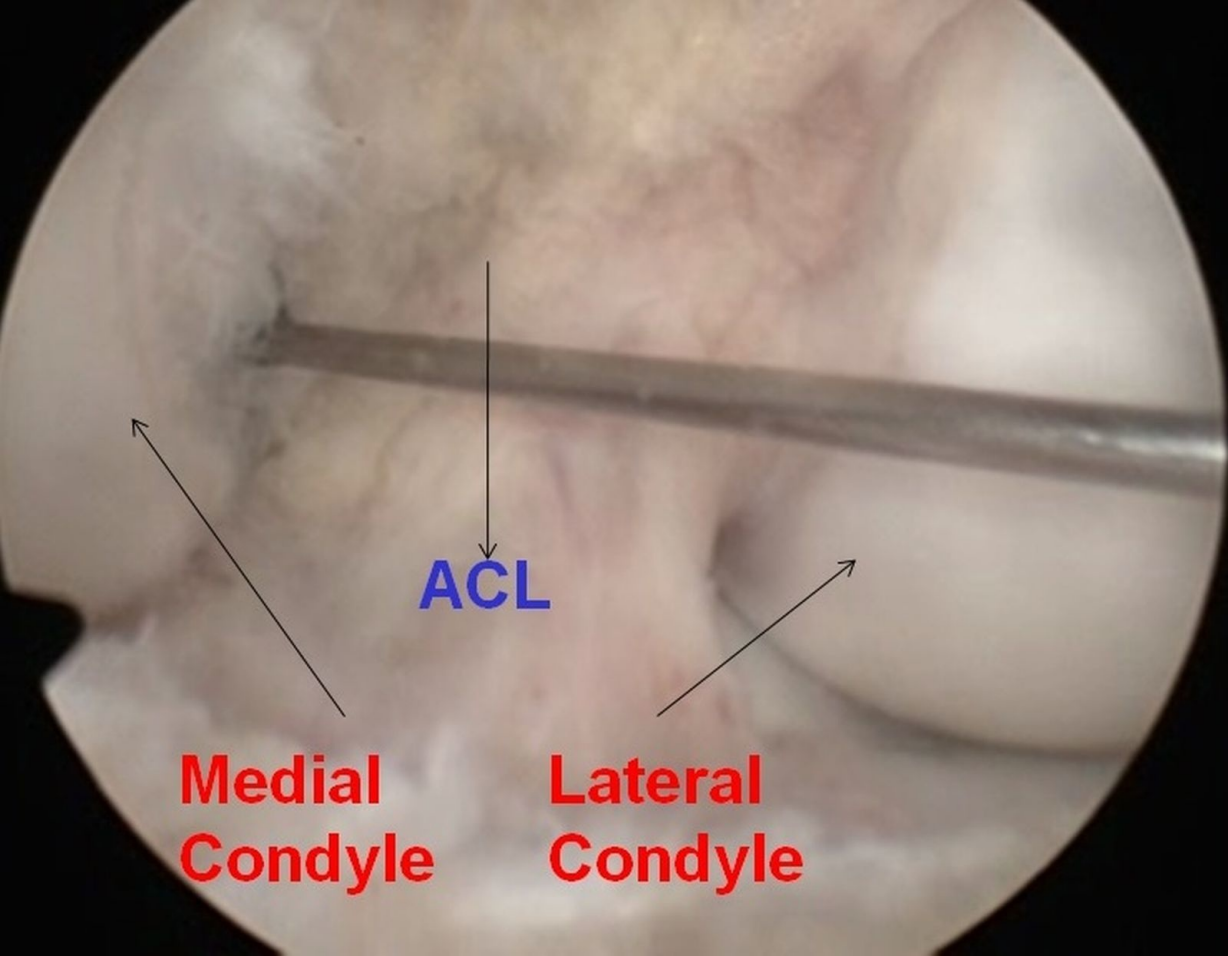
Arthroscopic picture showing insertion of guide wire over lesion

**Figure 5:**
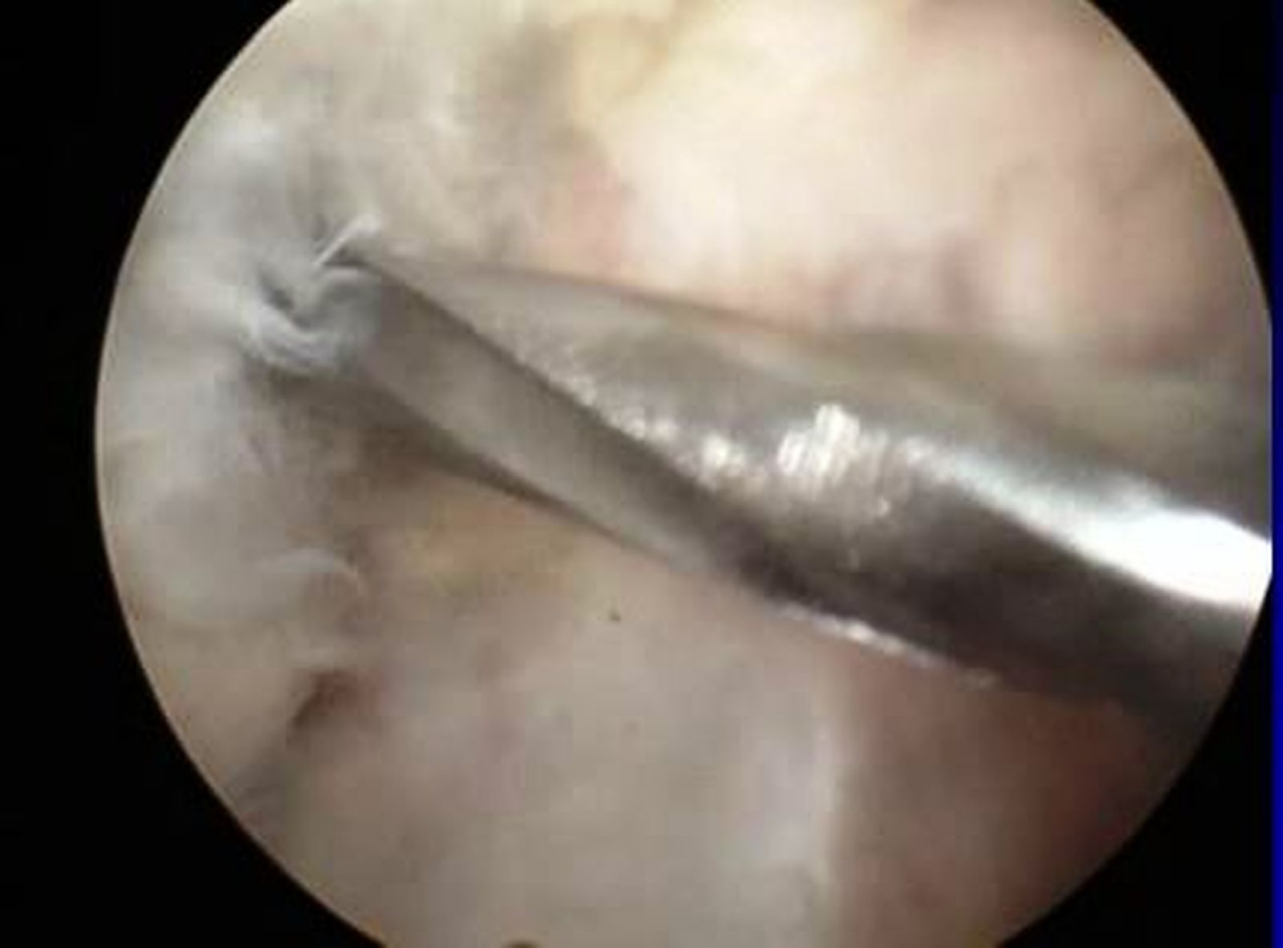
Arthroscopic picture showing evacuation of abscess and cavity with arthroscopic burr

Since signs of synovitis were present, arthroscopically assisted drainage was planned. The lesion was located on the lateral aspect of the medial condyle and was easily approachable using the arthroscope. Standard anterolateral and anteromedial portals were made. A guide wire was passed into the abscess cavity (Figure 4) via the anteromedial portal, and the position was checked using fluoroscopy. Then a cannulated drill was passed over the guide wire to decompress the cavity (Figure 5). Pus was seen extruding from the cavity. An arthroscopic burr was used to clean the cavity wall thoroughly.

Postoperatively, intravenous antibiotics were given for eight days followed by oral antibiotics for another two weeks. Physiotherapy was started from the third postoperative day. The drain was removed after 72 hours, and the patient was discharged on the eighth postoperative day. At the time of discharge, knee ROM was zero to 45 degrees, and at the one-month follow-up, knee ROM was zero to 90 degrees. At the two-year follow-up, there was no pain, with ROM from zero to 120 degrees.

## Discussion

Arthroscopic-assisted drainage of Brodie’s abscess of the distal femur is a minimally invasive procedure with various advantages like minimal blood loss, decreased chances of soft tissue complications, and early rehabilitation. However, it is not a suitable procedure in the presence of large lesions, which require curettage and bone grafting.

It has been suggested that surgery should be reserved for aggressive lesions. In the case of aggressive subacute osteomyelitis with an erythrocyte sedimentation rate (ESR) higher than 40 mm/hr, an abscess larger than 3 cm, or a lesion indistinguishable from a tumor, open biopsy for culture and histology is indicated [6]. Other lesions are incised and drained when indicated, and the granulation tissue present in the lesions is curetted. Antibiotics are started immediately after culture sensitivity analysis. In pediatric patients with typical cavities of the metaphysis, the epiphysis, or both, surgery is undertaken only for specific indications. When clinical signs of subperiosteal pus are present, incision and drainage are performed. When clinical signs of synovitis are present, with a possibility of pus within a joint, arthrotomy is performed, and synovium is sent for culture and histology studies.

If metaphyseal or epiphyseal cavities communicate with the joint, they are curetted. Curettage of cavities is also indicated if the symptoms and signs of infection persist during conservative treatment or if they recur. Curettage of metaphyseal cavities should be carried out carefully. The perforation in the growth plate should not be curetted because curettage of the metaphyseal lesion usually decompresses the epiphyseal lesion. Ross and Cole reported that all epiphyseal cavities in their study healed with a single course of antibiotics and immobilization without operation [7]. However, when drainage was indicated, the procedure was not performed through the growth plate.

Green et al. described curetting epiphyseal lesions after localization by inserting a needle into the epiphysis and obtaining two plain radiographs. A 3-mm drill hole is made in the epiphysis avoiding the weight-bearing or articulating portion [8]. In the proximal femoral epiphysis, the drill hole must be intracapsular as far distally as possible to avoid the part of the femoral head that articulates with the acetabulum, while avoiding the growth plate. In the distal femoral epiphysis, the drill hole also has to be intra-articular but avoid the weight-bearing articular surface coming medially or laterally.

## Conclusions

Arthroscopically assisted drainage of Brodie’s abscess has never been reported in the literature and is a useful, minimally invasive technique leading to an early recovery and faster rehabilitation.
